# Correction: Random Wiring, Ganglion Cell Mosaics, and the Functional Architecture of the Visual Cortex

**DOI:** 10.1371/journal.pcbi.1004758

**Published:** 2016-02-03

**Authors:** The PLOS Computational Biology Staff

There are several errors in Table 1. The authors confirm that the errors do not affect their conclusions in any way. Please view the correct version of Table 1 here:

**Table 1:** The six orientation domain layout parameters characterizing the common design.

	Pinwheel density ρ	NN distance ind. charge	NN distance same charge	NN distance opp. charge	Variab. Exponent γ	Variab. coeff. c
Ferret	3.14 [3.06, 3.23]	0.355 [0.347, 0.363]	0.530 [0.521, 0.539]	0.394 [0.383, 0.403]	0.40 [0.37, 0.44]	1.07 [0.97, 1.15]
Dark-reared Ferret	3.30 [3.16, 3.42]	0.346 [0.334, 0.361]	0.511 [0.499, 0.528]	0.381 [0.366, 0.401]	0.39 [0.35, 0.46]	1.02 [0.90, 1.12]
Cat	3.24 [3.06, 3.42]	0.366 [0.352, 0.381]	0.534 [0.519, 0.551]	0.407 [0.388, 0.428]	0.48 [0.41, 0.58]	0.83 [0.68, 0.95]
Treeshrew	3.08 [2.99 3.16]	0.364 [0.359 0.370]	0.521 [0.514 0.528]	0.404 [0.396 0.411]	0.36 [0.34 0.39]	1.13 [1.05 1.19]
Galago	3.12 [2.93, 3.27]	0.363 [0.345, 0.381]	0.536 [0.522, 0.556]	0.396 [0.375, 0.417]	0.45 [0.42, 0.52]	0.85 [0.71, 0.99]
Ensemble Average	3.13	0.359	0.525	0.397	0.40	1.05
Common Design–CR	[3.09 3.19]	[0.354 0.363]	[0.520 0.530]	[0.391 0.403]	[0.37 0.42]	[0.99 1.11]
One Species–CR	[2.93, 3.42]	[0.334, 0.381]	[0.499, 0.556]	[0.366, 0.428]	[0.34, 0.58]	[0.68, 1.19]

Values were calculated with the code provided in the supplemental material and intervals indicate 95% bootstrap confidence intervals. Also shown is the grand average and the associated one species and common design consistency ranges (CR).

doi:10.1371/journal.pcbi.1004602.t001
