# Supplementary figures and images for: Random Wiring, Ganglion Cell Mosaics, and the Functional Architecture of the Visual Cortex
Source: PLoS Comput Biol. 2015 Nov 17;11(11):e1004602. doi: 10.1371/journal.pcbi.1004602 (PMC4648540; doi:10.1371/journal.pcbi.1004602)

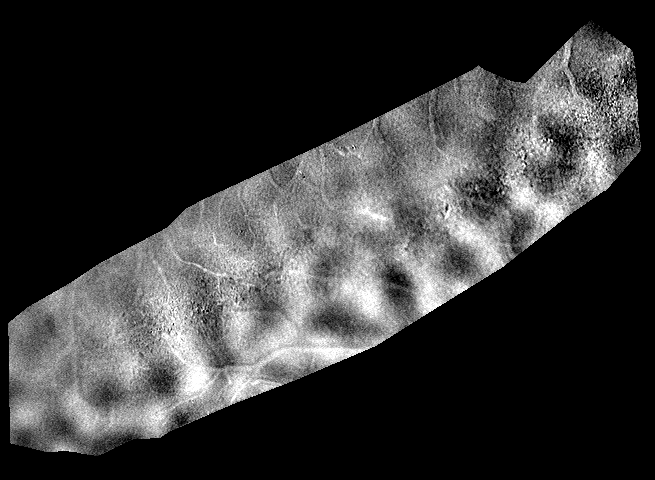

Supplement: S1 Code — We provide all necessary code to calculate single neuron properties and orientation domain layouts along with a Mathematica program which contains the analytical solution for both, an example single neuron and the domain layout obtained from a perfect and infinite lattice. The C-program ‘calculate_single_neuron.cpp’ calculates the same for a single neuron numerically. The C-program is provided to illustrate the use of the rfanalyzer class. The c-program ‘calculate_map.cpp’ calculates the same properties as ‘calculate_single_neuron.cpp’ but for a whole array of cells. After finishing a run, this program generates a set of ascii files in which the output is stored. These files are read in and analyzed by the Matlab program ‘plot_results.m’. It calculates the pinwheel density, pinwheel distance distributions, mean pinwheel distance and pinwheel density fluctuations as a function of subregion size. We compiled the code with gcc [g++ (Ubuntu 4.8.2-19ubuntu1) 4.8.2] and the gsl: g++ ./calculate_single_neuron.cpp -lgsl -lgslcblas -O3 -march = native g++ ./calculate_map.cpp -lgsl -lgslcblas -O3 -march = native For this article, we have calculated orientation domain layouts with aspect ratio 22x22Λ, sampled with 4096x4096 pixels. This corresponds to ≈ 6.5μm per cortical unit for our standard combination of parameters (r = r′ = 170 μm and Δα = 7°). Experimental data The folder ‘map_data’ contains a data folder with single condition layouts and various ROIs for four ferrets cases. It also contains two Matlab files, ‘run_analysis.m’ and ‘plot_results.m’ to run the analysis and display the results. The full data set used in the present study is available on the neural data sharing platform http://www.g-node.org/. (ZIP) [file pcbi.1004602.s001.zip › map_data/data/F112_lh/raw_difference_images/0-90.tif]

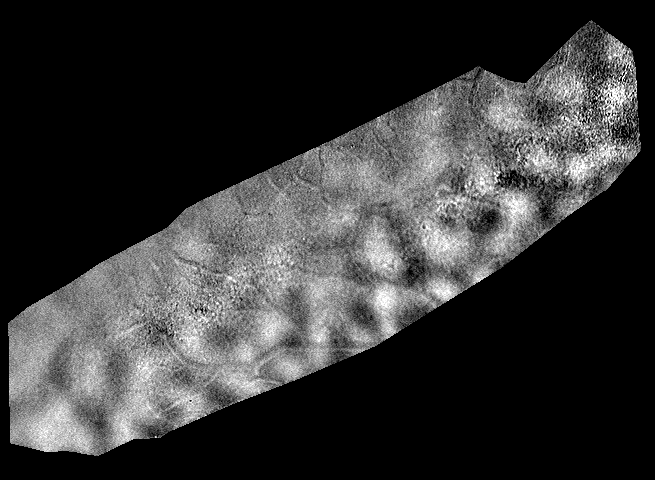

Supplement: S1 Code — We provide all necessary code to calculate single neuron properties and orientation domain layouts along with a Mathematica program which contains the analytical solution for both, an example single neuron and the domain layout obtained from a perfect and infinite lattice. The C-program ‘calculate_single_neuron.cpp’ calculates the same for a single neuron numerically. The C-program is provided to illustrate the use of the rfanalyzer class. The c-program ‘calculate_map.cpp’ calculates the same properties as ‘calculate_single_neuron.cpp’ but for a whole array of cells. After finishing a run, this program generates a set of ascii files in which the output is stored. These files are read in and analyzed by the Matlab program ‘plot_results.m’. It calculates the pinwheel density, pinwheel distance distributions, mean pinwheel distance and pinwheel density fluctuations as a function of subregion size. We compiled the code with gcc [g++ (Ubuntu 4.8.2-19ubuntu1) 4.8.2] and the gsl: g++ ./calculate_single_neuron.cpp -lgsl -lgslcblas -O3 -march = native g++ ./calculate_map.cpp -lgsl -lgslcblas -O3 -march = native For this article, we have calculated orientation domain layouts with aspect ratio 22x22Λ, sampled with 4096x4096 pixels. This corresponds to ≈ 6.5μm per cortical unit for our standard combination of parameters (r = r′ = 170 μm and Δα = 7°). Experimental data The folder ‘map_data’ contains a data folder with single condition layouts and various ROIs for four ferrets cases. It also contains two Matlab files, ‘run_analysis.m’ and ‘plot_results.m’ to run the analysis and display the results. The full data set used in the present study is available on the neural data sharing platform http://www.g-node.org/. (ZIP) [file pcbi.1004602.s001.zip › map_data/data/F112_lh/raw_difference_images/45-135.tif]

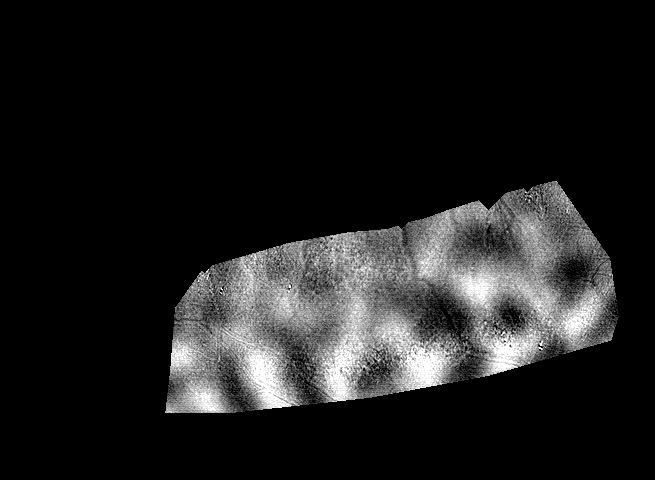

Supplement: S1 Code — We provide all necessary code to calculate single neuron properties and orientation domain layouts along with a Mathematica program which contains the analytical solution for both, an example single neuron and the domain layout obtained from a perfect and infinite lattice. The C-program ‘calculate_single_neuron.cpp’ calculates the same for a single neuron numerically. The C-program is provided to illustrate the use of the rfanalyzer class. The c-program ‘calculate_map.cpp’ calculates the same properties as ‘calculate_single_neuron.cpp’ but for a whole array of cells. After finishing a run, this program generates a set of ascii files in which the output is stored. These files are read in and analyzed by the Matlab program ‘plot_results.m’. It calculates the pinwheel density, pinwheel distance distributions, mean pinwheel distance and pinwheel density fluctuations as a function of subregion size. We compiled the code with gcc [g++ (Ubuntu 4.8.2-19ubuntu1) 4.8.2] and the gsl: g++ ./calculate_single_neuron.cpp -lgsl -lgslcblas -O3 -march = native g++ ./calculate_map.cpp -lgsl -lgslcblas -O3 -march = native For this article, we have calculated orientation domain layouts with aspect ratio 22x22Λ, sampled with 4096x4096 pixels. This corresponds to ≈ 6.5μm per cortical unit for our standard combination of parameters (r = r′ = 170 μm and Δα = 7°). Experimental data The folder ‘map_data’ contains a data folder with single condition layouts and various ROIs for four ferrets cases. It also contains two Matlab files, ‘run_analysis.m’ and ‘plot_results.m’ to run the analysis and display the results. The full data set used in the present study is available on the neural data sharing platform http://www.g-node.org/. (ZIP) [file pcbi.1004602.s001.zip › map_data/data/F123/raw_difference_images/0-90.tif]

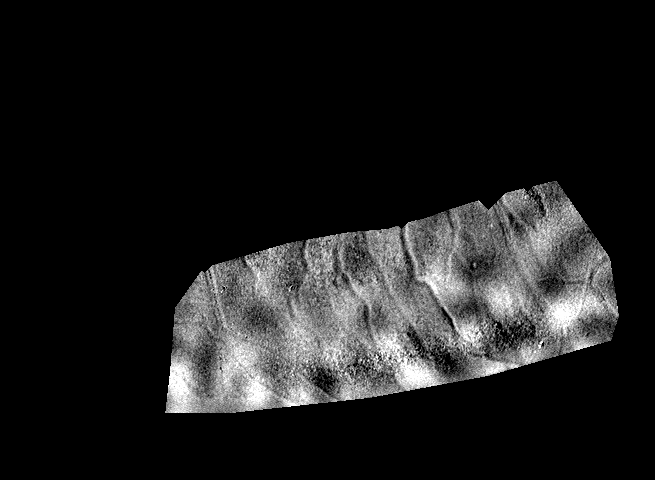

Supplement: S1 Code — We provide all necessary code to calculate single neuron properties and orientation domain layouts along with a Mathematica program which contains the analytical solution for both, an example single neuron and the domain layout obtained from a perfect and infinite lattice. The C-program ‘calculate_single_neuron.cpp’ calculates the same for a single neuron numerically. The C-program is provided to illustrate the use of the rfanalyzer class. The c-program ‘calculate_map.cpp’ calculates the same properties as ‘calculate_single_neuron.cpp’ but for a whole array of cells. After finishing a run, this program generates a set of ascii files in which the output is stored. These files are read in and analyzed by the Matlab program ‘plot_results.m’. It calculates the pinwheel density, pinwheel distance distributions, mean pinwheel distance and pinwheel density fluctuations as a function of subregion size. We compiled the code with gcc [g++ (Ubuntu 4.8.2-19ubuntu1) 4.8.2] and the gsl: g++ ./calculate_single_neuron.cpp -lgsl -lgslcblas -O3 -march = native g++ ./calculate_map.cpp -lgsl -lgslcblas -O3 -march = native For this article, we have calculated orientation domain layouts with aspect ratio 22x22Λ, sampled with 4096x4096 pixels. This corresponds to ≈ 6.5μm per cortical unit for our standard combination of parameters (r = r′ = 170 μm and Δα = 7°). Experimental data The folder ‘map_data’ contains a data folder with single condition layouts and various ROIs for four ferrets cases. It also contains two Matlab files, ‘run_analysis.m’ and ‘plot_results.m’ to run the analysis and display the results. The full data set used in the present study is available on the neural data sharing platform http://www.g-node.org/. (ZIP) [file pcbi.1004602.s001.zip › map_data/data/F123/raw_difference_images/45-135.tif]

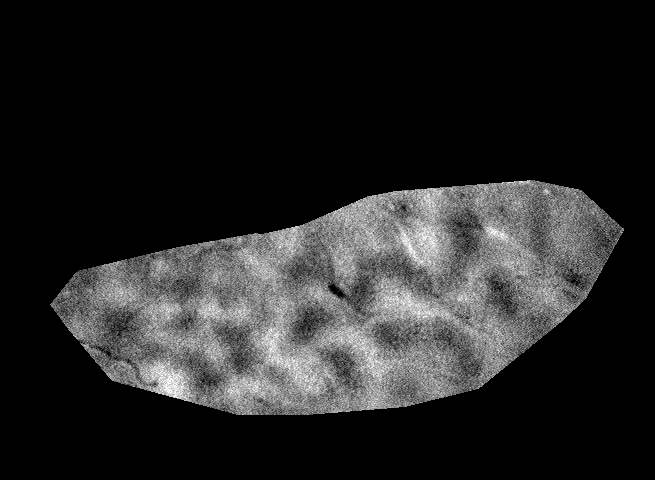

Supplement: S1 Code — We provide all necessary code to calculate single neuron properties and orientation domain layouts along with a Mathematica program which contains the analytical solution for both, an example single neuron and the domain layout obtained from a perfect and infinite lattice. The C-program ‘calculate_single_neuron.cpp’ calculates the same for a single neuron numerically. The C-program is provided to illustrate the use of the rfanalyzer class. The c-program ‘calculate_map.cpp’ calculates the same properties as ‘calculate_single_neuron.cpp’ but for a whole array of cells. After finishing a run, this program generates a set of ascii files in which the output is stored. These files are read in and analyzed by the Matlab program ‘plot_results.m’. It calculates the pinwheel density, pinwheel distance distributions, mean pinwheel distance and pinwheel density fluctuations as a function of subregion size. We compiled the code with gcc [g++ (Ubuntu 4.8.2-19ubuntu1) 4.8.2] and the gsl: g++ ./calculate_single_neuron.cpp -lgsl -lgslcblas -O3 -march = native g++ ./calculate_map.cpp -lgsl -lgslcblas -O3 -march = native For this article, we have calculated orientation domain layouts with aspect ratio 22x22Λ, sampled with 4096x4096 pixels. This corresponds to ≈ 6.5μm per cortical unit for our standard combination of parameters (r = r′ = 170 μm and Δα = 7°). Experimental data The folder ‘map_data’ contains a data folder with single condition layouts and various ROIs for four ferrets cases. It also contains two Matlab files, ‘run_analysis.m’ and ‘plot_results.m’ to run the analysis and display the results. The full data set used in the present study is available on the neural data sharing platform http://www.g-node.org/. (ZIP) [file pcbi.1004602.s001.zip › map_data/data/F236/raw_difference_images/0-90.tif]

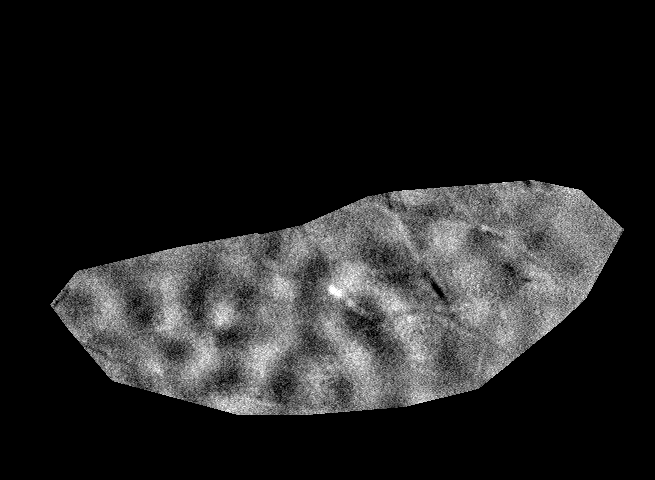

Supplement: S1 Code — We provide all necessary code to calculate single neuron properties and orientation domain layouts along with a Mathematica program which contains the analytical solution for both, an example single neuron and the domain layout obtained from a perfect and infinite lattice. The C-program ‘calculate_single_neuron.cpp’ calculates the same for a single neuron numerically. The C-program is provided to illustrate the use of the rfanalyzer class. The c-program ‘calculate_map.cpp’ calculates the same properties as ‘calculate_single_neuron.cpp’ but for a whole array of cells. After finishing a run, this program generates a set of ascii files in which the output is stored. These files are read in and analyzed by the Matlab program ‘plot_results.m’. It calculates the pinwheel density, pinwheel distance distributions, mean pinwheel distance and pinwheel density fluctuations as a function of subregion size. We compiled the code with gcc [g++ (Ubuntu 4.8.2-19ubuntu1) 4.8.2] and the gsl: g++ ./calculate_single_neuron.cpp -lgsl -lgslcblas -O3 -march = native g++ ./calculate_map.cpp -lgsl -lgslcblas -O3 -march = native For this article, we have calculated orientation domain layouts with aspect ratio 22x22Λ, sampled with 4096x4096 pixels. This corresponds to ≈ 6.5μm per cortical unit for our standard combination of parameters (r = r′ = 170 μm and Δα = 7°). Experimental data The folder ‘map_data’ contains a data folder with single condition layouts and various ROIs for four ferrets cases. It also contains two Matlab files, ‘run_analysis.m’ and ‘plot_results.m’ to run the analysis and display the results. The full data set used in the present study is available on the neural data sharing platform http://www.g-node.org/. (ZIP) [file pcbi.1004602.s001.zip › map_data/data/F236/raw_difference_images/45-135.tif]

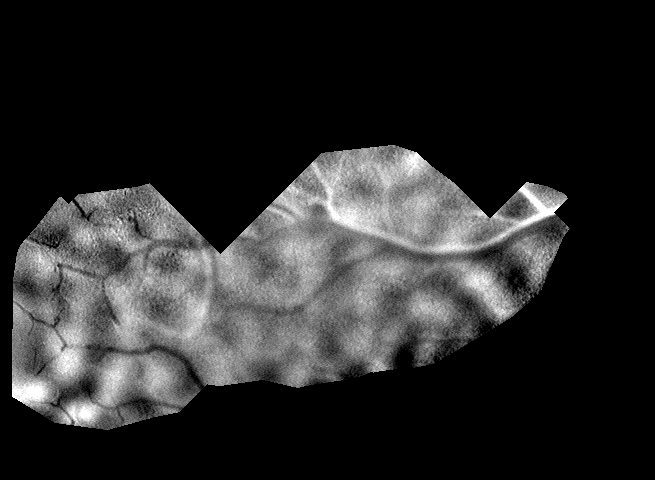

Supplement: S1 Code — We provide all necessary code to calculate single neuron properties and orientation domain layouts along with a Mathematica program which contains the analytical solution for both, an example single neuron and the domain layout obtained from a perfect and infinite lattice. The C-program ‘calculate_single_neuron.cpp’ calculates the same for a single neuron numerically. The C-program is provided to illustrate the use of the rfanalyzer class. The c-program ‘calculate_map.cpp’ calculates the same properties as ‘calculate_single_neuron.cpp’ but for a whole array of cells. After finishing a run, this program generates a set of ascii files in which the output is stored. These files are read in and analyzed by the Matlab program ‘plot_results.m’. It calculates the pinwheel density, pinwheel distance distributions, mean pinwheel distance and pinwheel density fluctuations as a function of subregion size. We compiled the code with gcc [g++ (Ubuntu 4.8.2-19ubuntu1) 4.8.2] and the gsl: g++ ./calculate_single_neuron.cpp -lgsl -lgslcblas -O3 -march = native g++ ./calculate_map.cpp -lgsl -lgslcblas -O3 -march = native For this article, we have calculated orientation domain layouts with aspect ratio 22x22Λ, sampled with 4096x4096 pixels. This corresponds to ≈ 6.5μm per cortical unit for our standard combination of parameters (r = r′ = 170 μm and Δα = 7°). Experimental data The folder ‘map_data’ contains a data folder with single condition layouts and various ROIs for four ferrets cases. It also contains two Matlab files, ‘run_analysis.m’ and ‘plot_results.m’ to run the analysis and display the results. The full data set used in the present study is available on the neural data sharing platform http://www.g-node.org/. (ZIP) [file pcbi.1004602.s001.zip › map_data/data/F76/raw_difference_images/0-90.tif]

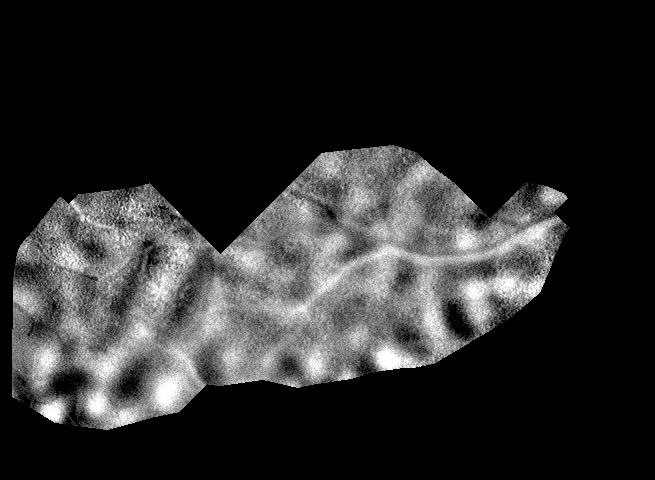

Supplement: S1 Code — We provide all necessary code to calculate single neuron properties and orientation domain layouts along with a Mathematica program which contains the analytical solution for both, an example single neuron and the domain layout obtained from a perfect and infinite lattice. The C-program ‘calculate_single_neuron.cpp’ calculates the same for a single neuron numerically. The C-program is provided to illustrate the use of the rfanalyzer class. The c-program ‘calculate_map.cpp’ calculates the same properties as ‘calculate_single_neuron.cpp’ but for a whole array of cells. After finishing a run, this program generates a set of ascii files in which the output is stored. These files are read in and analyzed by the Matlab program ‘plot_results.m’. It calculates the pinwheel density, pinwheel distance distributions, mean pinwheel distance and pinwheel density fluctuations as a function of subregion size. We compiled the code with gcc [g++ (Ubuntu 4.8.2-19ubuntu1) 4.8.2] and the gsl: g++ ./calculate_single_neuron.cpp -lgsl -lgslcblas -O3 -march = native g++ ./calculate_map.cpp -lgsl -lgslcblas -O3 -march = native For this article, we have calculated orientation domain layouts with aspect ratio 22x22Λ, sampled with 4096x4096 pixels. This corresponds to ≈ 6.5μm per cortical unit for our standard combination of parameters (r = r′ = 170 μm and Δα = 7°). Experimental data The folder ‘map_data’ contains a data folder with single condition layouts and various ROIs for four ferrets cases. It also contains two Matlab files, ‘run_analysis.m’ and ‘plot_results.m’ to run the analysis and display the results. The full data set used in the present study is available on the neural data sharing platform http://www.g-node.org/. (ZIP) [file pcbi.1004602.s001.zip › map_data/data/F76/raw_difference_images/45-135.tif]
